# DNA Damage Response in Plants: Conserved and Variable Response Compared to Animals

**DOI:** 10.3390/biology2041338

**Published:** 2013-11-21

**Authors:** Kaoru Okamoto Yoshiyama, Kengo Sakaguchi, Seisuke Kimura

**Affiliations:** 1 Department of Bioresource and Environmental Sciences, Kyoto Sangyo University, Kamigamo-Motoyama, Kitaku, Kyoto 603-8555, Japan; 2 Research Institute for Science and Technology, Tokyo University of Science, 2641 Yamazaki, Noda, Chiba 278-8510, Japan; E-Mail: kengo@rs.noda.tus.ac.jp

**Keywords:** DNA damage response, genome stability, signaling, Arabidopsis, SOG1, p53

## Abstract

The genome of an organism is under constant attack from endogenous and exogenous DNA damaging factors, such as reactive radicals, radiation, and genotoxins. Therefore, DNA damage response systems to sense DNA damage, arrest cell cycle, repair DNA lesions, and/or induce programmed cell death are crucial for maintenance of genomic integrity and survival of the organism. Genome sequences revealed that, although plants possess many of the DNA damage response factors that are present in the animal systems, they are missing some of the important regulators, such as the p53 tumor suppressor. These observations suggest differences in the DNA damage response mechanisms between plants and animals. In this review the DNA damage responses in plants and animals are compared and contrasted. In addition, the function of SUPPRESSOR OF GAMMA RESPONSE 1 (SOG1), a plant-specific transcription factor that governs the robust response to DNA damage, is discussed.

## 1. Introduction

The genome contains all the necessary information required for the development and maintenance of an organism, which is why it is important to protect the DNA from damage caused by the action of exogenous (e.g., ionizing radiation (IR), ultraviolet (UV), and chemical mutagens) and endogenous (e.g., metabolic byproducts, stalled replication forks) sources. In response to DNA damage, eukaryotic cells activate elaborate cellular networks, collectively termed the DNA damage response (DDR), which is critical for maintaining genome integrity [1]. These signaling pathways lead to DNA repair, cell-cycle arrest (cell-cycle checkpoint), and, eventually, apoptosis to remove or tolerate lesions in their genetic material. When DNA damage is not severe, cell-cycle progression is delayed or arrested to gain time for repairing the damage. On the other hand, when DNA damage is severe, animal cells undergo apoptosis, as it is preferable to eliminate cells with unrepairable DNA than to allow them to propagate incorrect genetic information. Therefore, DDR is not only a fundamental cellular process for protecting cells from the DNA damage but is also indispensable in ensuring faithful transmission of genetic information from one generation to the next.

In contrast to most animals, plants are sessile organisms that cannot change their location. As plants require light-containing UV for their photosynthetic activity and as their chloroplasts continuously generate reactive oxygen species (ROS) [2,3,4], plants are believed to be constantly exposed to DNA damage. Moreover, in contrast to animal development, plant development is mostly a postembryonic process, which is achieved by the activity of meristems, in which cells divide throughout the plant’s life [5]. It is, thus, imperative that an efficient and specific DDR system be in place in plants to cope with DNA damage. Therefore, it would be important to understand the mechanisms of plant DDR system, but to date, these mechanisms have mainly been investigated in yeasts and animals, and only recently have plant DDR systems begun to be studied in detail.

The completion of genome sequencing for several plants has accelerated the study of the DDR in plants. Many homologs of the evolutionarily conserved DDR components have been reported in plant genomes [6]. Although the basic regulatory mechanisms are conserved in other eukaryotes, plants have evolved new regulators of DDR. Presence of these plant-specific genes suggests a unique DDR system in plants. In this review, we have compared and contrasted the DDR mechanisms between animals and plants. In particular, we have highlighted a plant-specific DDR transcription factor, SUPPRESSOR OF GAMMA RESPONSE 1 (SOG1).

## 2. Components of the DDR Pathway in Animals and Plants

**Figure 1:**
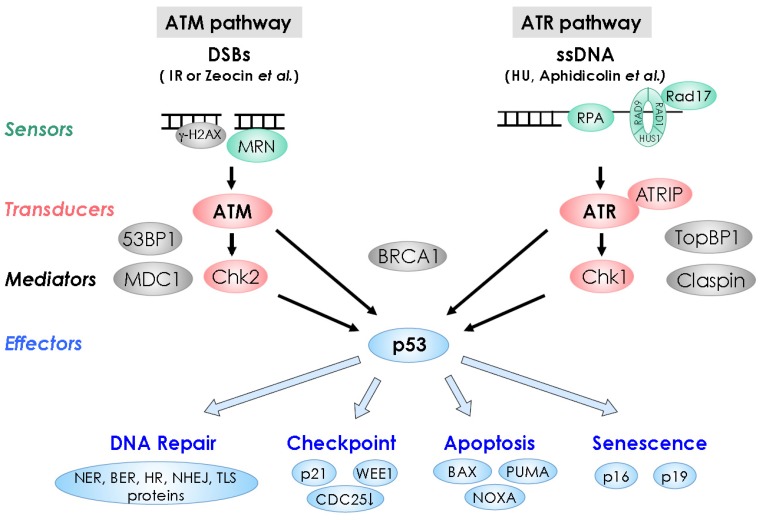
DNA damage response pathways in animals.Double-strand DNA break (DSB) and single-stranded DNA signal through the sensors shown in green, signal transducing kinases shown in red, mediators shown in gray, and effectors shown in blue, leading to DNA repair, cell-cycle checkpoint, apoptosis, or senescence.

In mammals, members of the phosphoinositide 3-kinase-like kinase (PIKK) family, such as ataxia telangiectasia mutated (ATM), ATM and Rad3-related (ATR), and DNA-dependent protein kinase catalytic subunit (DNA-PKcs) are rapidly activated in response to DNA damage [7]. Among them, ATM and ATR are the major regulators of the DDR. ATM responds to DNA double-strand breaks (DSBs) (ATM pathway), while ATR responds to a wide range of DNA lesions, especially those associated with DNA replication (ATR pathway) [8]. At the molecular level, the DDR pathway contains several key components: DNA damage sensors, signal transducers, mediators, and effectors (Figure 1), and most of them were first identified and studied in yeasts and animals [9]. In this review, we have discussed some of the major regulators involved in animal and plant DDRs.

### 2.1. DNA Damage Sensors

Various types of DNA damage can be detected by distinct sensor proteins. In mammals, the MRE11/RAD50/NBS1 (MRN) complex has been proposed as DSB sensors in the ATM pathway [10]. The MRN complex recruits ATM at the DSB sites, and the interaction with NBS1 activates ATM kinase, resulting in the phosphorylation of the target proteins. One of the earliest consequences of ATM activation at the DSB site is phosphorylation of the histone-variant H2AX producing γH2AX [11]. γH2AX acts as a signal for DNA damage and recruits the DDR proteins. Replication protein A (RPA), a single-strand DNA-binding protein, functions as a sensor in the ATR pathway [12]. RPA binds to single-stranded DNA (ssDNA) generated at the site of stalled replication forks. ATR-interacting protein (ATRIP) binds to the RPA-ssDNA complex in order to recruit ATR to ssDNA, which is followed by the activation of the checkpoint kinase CHK1 [12]. The RAD9/RAD1/HUS1 (9-1-1) complex is also a DNA-damage sensor and it activates the ATR pathway. The 9-1-1 complex forms a ring-like clamp similar to that of a proliferating cell nuclear antigen (PCNA) homotrimer [13,14]. RAD17 acts as a clamp loader responsible for the loading of the 9-1-1 complex [13].

In *Arabidopsis thaliana*, one of the earliest responses to IR-induced DSBs is also the phosphorylation of the histone-variant H2AX (γH2AX). The formation of γH2AX foci is dependent on the kinase activity of AtATM (*A. thaliana* homolog of mammal ATM) and AtATR (*A. thaliana* homolog of mammal ATR) [15]. Plant homologs of *MRE11*, *RAD50*, and *NBS1* have also been identified in *A. thaliana* [16,17,18]. γH2AX induction did not occur in *rad50* or *mre11* mutant plants, suggesting that the MRN complex is required for H2AX phosphorylation by the ATM and ATR kinases in response to DNA damage [19]. These observations suggest that the MRN complex acts as a sensor for the DDR, via conserved mechanisms both in plants and animals. 

Inactivation of *A. thaliana* RPA70a, which is similar to the largest subunit of human RPA, causes increased sensitivity to the replication stress agent hydroxyurea (HU), suggesting a role for AtRPA in the ATR pathway [20]. Homologs of genes of the 9-1-1 complex and RAD17 are also found in *A. thaliana* genome, and they share conserved sequence domains with their human counterparts [21]. The difficulty in controlling cell cycle in cultured plant cells has discouraged extensive and detailed functional analysis of cell-cycle regulation in *A. thaliana*. However, both *rad17* and *rad9* mutants of *A. thaliana* are sensitive to bleomycin (BLM) and mitomycin C (MMC) [21] and have similar phenotypes to those of mutants for the corresponding human genes. Furthermore, homozygous *rad9 rad17* double mutants have similar sensitivity to BLM and MMC as single mutants, suggesting that the two proteins are involved in the same pathway in plants as in animals. Considering these observations, it appears that the mechanisms for sensing DNA damage in the ATM and ATR pathways are conserved between animals and plants.

### 2.2. Signal Transducers

DNA damage sensors transmit signals to transducers, which then amplify and transduce signals to downstream effectors. In animals, ATM/ATR and the downstream CHK1/CHK2 are well-studied transducers in the DDR pathway. These are serine/threonine kinases that initiate a cascade of phosphorylation events. Following exposure to IR, ATM is activated through autophosphorylation of ATM and monomerization of the ATM dimer [22]. ATR is recruited to RPA-coated ssDNA lesions by the ATRIP [23,24]. Both ATM and ATR phosphorylate SQ/TQ motifs and share substrates, including breast cancer susceptibility gene 1(BRCA1), NBS1, p53, CHK1, and CHK2 [25]. In mammals, knockout ATR mutation results in embryonic lethality [26], while an ATM mutation results in pleiotrobic defects (e.g., growth defects, neurologic dysfunction, and infertility) [27]. CHK1 and CHK2 are the key transducers that receive signal from ATR and ATM, respectively [28,29]. In response to DNA damage, CHK1 and CHK2 are phosphorylated and activated in an ATM/ATR-dependent manner [30,31,32,33,34]. CHK1 and CHK2 also share many common substrates similar to ATM and ATR, such as BRCA1, p53, E2F1, and CDC25A [35].

AtATM and AtATR have been identified in *A. thaliana* [36,37]. In contrast to animals, *atm* and *atr* mutants are both phenotypically normal, except for a partial sterility in the *atm* mutant. The *atm* mutant is sensitive to DSB-inducing agents (e.g., IR and methyl methanesulfonate) [36], and the *atr* mutant is sensitive to replication stress-inducing agents (e.g., aphidicolin or HU) [37]. These phenotypic analyses indicate conservation of the roles of these proteins in plants. Furthermore, *A. thaliana atm* mutants fail to induce transcriptional upregulation of many genes following IR [38]. As microarray analysis in human cells has demonstrated that ATM plays a critical role in regulating gene expression in response to DNA damage [39,40], the plant *atm* mutant phenotype is consistent with the role of ATM in DNA damage signaling in plants. ATRIP ortholog is also found in *A. thaliana* [41,42]. Although no data demonstrate direct interaction with ATR and ATRIP, null-mutant lines of ATR and ATRIP demonstrate the same phenotype in hypersensitivity to replication stress agents and IR. *atr atrip* double mutants do not possess significant additive sensitivity effects in response to replication blocks or IR [41,42], suggesting that ATR and ATRIP participate in the same genetic pathway. Apparently, *A. thaliana* have no CHK1 and CHK2 ortholog. As some of substrates of CHK1 and CHK2 in animals (e.g., BRCA1 and E2F) are present in plants [43,44], other kinases may work as functional homologs of CHK1 and CHK2.

### 2.3. Mediators

Mediator proteins work to coordinate the temporal-spatial regulation of the various factors in the DDR, promote their activation, recruit additional substrates, and control their association with damaged DNA [45,46]. Mediator of DNA-damage checkpoint protein 1 (MDC1), p53-binding protein (53BP1), and BRCA1 appear to be largely linked to the ATM pathway [47], whereas DNA topoisomerase 2-binding protein 1 (TOPBP1) and CLASPIN have been proposed to coregulate the ATR pathway [48,49]. A subgroup of mediators, BRCA1, 53BP1, MDC1, and TOPBP1, contain BRCA1 associated C-terminal (BRCT) domains, which interact with phosphorylated proteins [50,51]. CLASPIN interacts with ATR, CHK1, and BRCA1, and regulates CHK1 and BRCA1 phosphorylation following replication stress [52,53]. H2AX is also a mediator protein. H2AX is phosphorylated (γH2AX) by ATM and ATR upon DNA damage [54], and is required for the efficient retention of NBS1 and the other mediator proteins, BRCA1, MDC1, and 53BP1 at damaged DNA [46,55,56]. As MDC1 mediates γH2AX-ATM interaction, they form a positive feedback loop. Following the initial phosphorylation of H2AX at the sites of DNA damage, MDC1 leads to the accumulation of active ATM near the damaged DNA and results in the stimulation of ATM-dependent phosphorylation of H2AX [57]. These mediators function as scaffold proteins to create a platform for the recruitment of many tumor suppressors and DNA damage repair proteins involved in DDR signaling.

Although cancer does not develop in plants, a homolog of BRCA1 (AtBRCA1) is found in *A. thaliana* genomes [44]. AtBRCA1 also contains two C-terminal BRCT domains. Human BRCA1 and *A. thaliana* BRCA1 proteins showed 61% similarity in the BRCT region. The transcription of *AtBRCA1* is strongly induced by IR [38,44]. As *BRCA1* expression in mammals is similarly induced by IR [58], a similar regulatory mechanism for *BRCA1* expression after DNA damage in plants probably exists. However, AtBRCA1 carries a PHD domain (plant homeodomain) that is not present in animals and may be involved in the transcriptional regulation of plant development [59]. This observation hints at the possibility of AtBRCA1 acquiring plant-specific functions during evolution. Although *A. thaliana* MEIOSIS DEFECTIVE 1 (MEI1), which is involved in male meiosis, contains five BRCT domains and is similar to human TOPBP1 (25% identity, 40% similarity) [60], additional analysis is required to confirm that this protein works as a mediator. Other mediators, such as MDC1, 53BP1, and CLASPIN found in animal have not yet been identified in plants. These mediator proteins may be absent in plants.

### 2.4. Effectors

Signals from transducers activate downstream effectors, and the effectors elicit appropriate responses. The most important effector in animals is the p53 transcription, which functions as a tumor suppressor [61]. p53 plays a central role in the decision of a cell to either undergo cell-cycle arrest and DNA repair or apoptosis after DNA damage in animals [62]. The amount and transcriptional activity of p53 is regulated by post-translational modifications such as phosphorylation, sumoylation, neddylation, and acetylation [63,64]. In normal cells, the p53 protein levels are low because of MDM2-mediated ubiquitination and degradation through the proteasome pathway. Upon DNA damage, p53 is activated by phosphorylation at several N-terminal sites in its transactivation domain by ATM, ATR, CHK1, and CHK2 [62,65], and the phosphorylation site inhibits the interaction of p53 with MDM2, resulting in p53 stabilization [66].

Interestingly, no plant homologs of p53 have been identified in any of the model plants, which is likely linked to the absence of the core apoptotic machinery in plants [67]. This observation raises the question of whether plants have a factor that has similar function with p53. An answer to this can be SOG1, which has been described in Section 3.

#### 2.4.1. Effectors for Cell-Cycle Arrest (Cell-Cycle Checkpoint)

The DNA repair pathway is tightly coordinated with cell-cycle progression through the activation of orchestrated signaling pathways termed as cell-cycle checkpoints [68,69,70]. In response to DNA damage, cell-cycle progression is delayed or arrested at critical stages before or during DNA replication and before cell division. In animals, cyclin-dependent kinase (CDK) inhibitors, p21 and WEE1 kinase, are important effectors to halt the cell cycle in response to DNA damage [71,72]. p21 is the primary regulator of p53-mediated G1 arrest [73], and WEE1 is the key inhibitor of mitotic entry [74,75]. CDC25 phosphatases (CDC25A, CDC25B, CDC25C) that remove inhibitory phosphorylation on CDK to control progression of cell cycle are also effectors for cell-cycle arrest [76]. After DNA-damage treatment, CHK1 and CHK2 kinases inactivate CDC25s functions by phosphorylation and lead to cell-cycle arrest [77]. Although no plant homologs of p21 have been identified yet, several CDK inhibitors have been found in *A. thaliana*. These proteins fall into two families: One is Kip-related protein (KRP) family and the other is *SIAMESE/SIAMESE-RELATED* (*SIM/SMR*) family. Because *SMR4* (AT5G02220) and *SMR5* (AT1G07500), are strongly induced by treatment of DSBs induced agents [38,78], these SMR proteins might be involved in cell-cycle arrest in response to DNA damage. *A. thaliana* has a WEE1 homolog (AtWEE1) [79]. The *AtWEE1* is activated by DNA damage or by DNA-replication arrest in an AtATM- or AtATR-dependent manner, respectively [80]. AtWEE1-deficient plants do not accumulate phosphorylated AtCDKs and are sensitive to replication-stress agents. Furthermore, cell-cycle arrest was observed upon induction of the At*WEE1* expression, which indicates that AtWEE1 controls cell-cycle arrest in the DDR pathway. A CDC25-like protein was identified in *A. thaliana*, but it only consists of a *C*-terminal catalytic domain of animals, CDC25s [81]. The phenotype of *cdc25-like* null mutants and the overexpression lines is comparable with the corresponding wild-type, implying that CDC25-like protein may not be involved in cell-cycle regulation [82].

#### 2.4.2. Effectors for DNA Repair

Several DNA repair mechanisms for responding to different types of DNA damage have been identified from fungi to animals [83]. p53 promotes genomic integrity by regulating various genes involved in the DNA repair pathways such as nucleotide excision repair (NER), base excision repair (BER), translesion synthesis (TLS), homologous recombination (HR), and non-homologous end-joining (NHEJ) [62]. Components of these DNA repair pathways are mostly conserved between animals and plants [84,85], suggesting that animals and plants have similar DNA repair mechanisms. Both in animals and plants, NHEJ is active throughout the cell cycle and is favored in G1 cells. On the other hand, HR is restricted to G2/M because HR requires the presence of intact sister chromatid to promote repair. Therefore, transformed DNA integrates mainly via NHEJ in an undirected, sequence-independent manner in the genome. However, in animals, gene targeting is achieved with 10^−2^ or higher frequency (one homologous integration event per hundred random integration events) using embryonic stem cells [86]. Although gene targeting is very efficient in the moss, somehow the frequency of gene targeting is low, 10^−4^ to 10^−5^ in plants Tobacco protoplasts [87,88]. These observations may suggest that choice of DNA repair pathway is different between animals and plants.

#### 2.4.3. Effectors for Programmed Cell Death (PCD) or Apoptosis

In animals, apoptosis is an important pathway in the DDR, and it is more stringent than cell-cycle arrest or repair in order to reduce the risk of accumulating cells with a compromised genomes. p53 activates genes involved in apoptosis, such as *BAX*, *PUMA*, and *NOXA* [89]. It has been shown that DNA damage induces cell death in the stem cell and their immediate progeny positioned in the shoot apical meristem and in the root apical meristem in plants [90]. AtATM and AtATR are needed for the stem cell death. Although animal stem cells are also specifically susceptible to genotoxic stress [91], the stem cell death in *A. thaliana* was morphologically distinct from apoptosis in animals. The stem cell death is similar to PCD, which is the autolytic cell death observed in plant developmental processes [90]. Plant PCD pathways seem to use mechanisms that are different from those found in animals because of lack of core apoptotic machinery in plants [67]. However, when animal BAX is expressed in plants, it trigger cell death [92,93]. In addition, BAX inhibitor-1 (BI-1), cell death suppressor, is conserved in both animals and plants, and *Arabidopsis BI-1* functions as a cell death attenuator for both biotic and abiotic types of cell death [94]. Furthermore, although caspases, key initiator or executioner for apoptosis in animals, are missing in plants, metacaspases (MCs), structurally related to caspases, were found in plants [95]. Transgenic overexpression of some *Arabidopsis* MCs (*AtMC4*, *AtMC8*) increased the level of cell death induction upon treatment with ROS inducing agents [96]. Loss of those genes’ expression result in a decrease or a delay of cell death [96]. These results may imply that plants and animals share a similar cell-death pathway, and some of these machineries might be regulated by SOG1 when plants suffer DNA damage.

## 3. Plant-Specific DDR

### 3.1. Endoreduplication

Endoreduplication is replication of the nuclear genome without cell division, resulting in elevated genomic DNA content [97]. It has been recently reported that genotoxic stress promotes endoreduplication in both plants and animals, but the function of this response in animals is not clear because endoreduplication can block mitosis but can also promote cancer progression depending on genetic background and tissue environment [98]. In plants, endoreduplication is one of the common responses to DNA damage as well as cell-cycle arrest and cell death. DSBs caused by depletion of the chromatin assembly factor 1 lead to extra endoreduplication cycles during leaf development [99,100,101]. DNA damages generated by *ETG1* (component of the replisome) mutation stimulate endoreduplication in leaves [102]. Furthermore, treatment with the DNA-damaging drug zeocin significantly increases endoreduplication in leaves or roots of wild-type plants [100,103]. These results indicate that damaged proliferating cells other than stem cells exit the cell cycle by endoreduplication in response to DNA damage. The reason why plants induce endoreduplication in response to DNA damage is unclear. To prevent the progeny from DNA mutations, the damaged cells might be entered into a non-dividing state by endoreduplication. The size of cells is often associated with their DNA content [104]. Therefore, the cell enlargement induced by endoreduplication might be able to compensate a reduction of cell number in the damaged tissue to keep their growth and tissue structure during the life cycle.

### 3.2. Plant-Specific Factors of the DDR

A plant-specific DDR factor has been identified recently. *MAINTENANCE OF MERISTEMS* (*MAIN*) is required for the maintenance of stem cells through a reduction in DNA damage [105,106]. Plants without functional MAIN display hypersensitivity to DNA-damaging agents. In *main* mutants, the expression levels of the DNA damage-inducible genes *BRCA1* and *RAD51* are increased, suggesting that the absence of this gene induces endogeneous DNA damage. As this gene is plant-specific, it may be involved in the plant-specific DDR pathway. Further genetic and molecular studies are needed to elucidate the precise molecular function of MAIN. Wei *et al*. recently reported that small RNAs play an important role in efficient DSB repair pathway in plants [107]. AtATR is involved in this pathway; therefore, small RNAs are novel regulators of the DDR system. It would be interesting to uncover the molecular mechanisms of DSBs repair mediated by small RNAs. The function of small RNAs appears to be conserved in animals, but further analysis is required.

*A. thaliana* SOG1 is one of the NAC (NAM, ATAF1/2, and CUC2) proteins, which constitutes one of the largest families of plant-specific transcription factors [78]. *SOG1* was identified in a screening method for suppressor mutants of the IR-induced cell-cycle arrest of *Arabidopsis xpf-2*, which is defective for the repair endonuclease *XPF* [108]. SOG1 is the first identified plant-specific transcription factor involved in the DDR pathway. It is also the central regulator of the DDRs because its activation is required for the majority of the plant’s responses to DNA damage, including transcriptional response, cell-cycle arrest, and death of stem cells.

#### 3.2.1. SOG1 Function

Recently we showed that SOG1 functions mainly in tissues that contain dividing cells, suggesting that SOG1 is required in actively dividing cells [109]. It has been reported that hundreds of genes are upregulated after IR, and it is completely abolished in *sog1-1* mutant, which indicates that a great majority of the transcriptional activation response to IR is regulated through SOG1 [78]. SOG1 is not only involved in the induction but also in the suppression of genes regulating cell-cycle progression. The expression of CDKB2;1 (a G2-associated transcript encoding a protein required for progression from G2 to M) and KNOLLE (a G2/M transcript encoding a protein required for cytokinesis) that promote cell-cycle progression was suppressed in the wild-type after IR, and this suppression was impaired in the *sog1-1* mutant. Furthermore, the *sog1-1* mutation enhanced IR-induced loss of heterozygosity, suggesting that the transcriptional response through SOG1 contributes to the maintenance of genomic stability after DNA damage [78]. Epidermal cells of the root tip entered the endoreduplication cycle by treatment with the radiomimetic reagent zeocin along with plant-specific CDKB2 protein degradation [103]; SOG1 is required in DNA damage-induced endoreduplication. In stem cells, SOG1 elicits PCD instead of endoreduplication after UV or IR treatment [110]. These observations suggest that SOG1 regulates different sets of genes depending on the cell types.

#### 3.2.2. The Mechanisms of SOG1 Regulation

**Figure 2:**
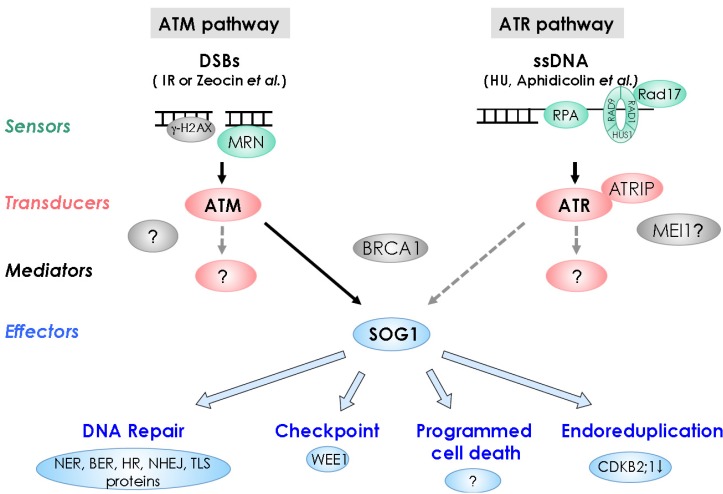
DNA damage response pathways in plants. DNA damage signal through the sensors shown in green, signal transducing kinases shown in red, mediators shown in gray, and effectors shown in blue, leading to DNA repair, cell-cycle checkpoint, programmed cell death, and endoreduplication. Dashed lines denote hypothetical situations.

We recently showed that SQ motif(s) of SOG1 is/are phosphorylated in an AtATM-dependent manner in response to DNA damage [109]. Unphosphorylated SOG1 mutant lost almost all SOG1 functions, indicating that the phosphorylation of SOG1 is indispensable for SOG1 functions. Analysis of SOG1 functions and the regulation mechanism demonstrates that the roles of SOG1 in the DDR system are comparable to those of animal p53 (Figure 2). As mentioned earlier, p53 is also a transcription factor, which is a critical component in the DDR system. p53 becomes activated upon DNA damage by ATM/CHK2 or ATR/CHK1 through phosphorylation of SQ motifs in the *N*-terminal region. Hence, SOG1 is a good candidate as a master regulator of the DDR in plants, and could thus be regarded as a functional homolog of p53. The only difference observed thus far is that the SOG1 function is not controlled by accumulation of proteins such as observed for p53 (Effectors section) [109]. The amino-acid sequences of SOG1 and p53 showed no similarity [109], suggesting that plants independently acquired plant-specific SOG1 during the evolutions of the DDR. 

Although p53 have conserved from multicellular invertebrates (insects, worm) to mammals [111], no orthologs have been identified in unicellular yeasts. The members of the NAC protein family to which SOG1 belongs, are present in a wide range of multicellular land plants, but are not in the unicellular green alga [78], suggesting that SOG1 might be conserved in multicellular plants, but not be conserved in unicellular green alga. In addition, it is reported that the target gene set for p53 are determined by tissue type [112]. As shown above, stem cells and epidermal cells of roots respond differently to DNA damage through the SOG1 pathway [90,103], implying that the target gene set for SOG1 may also be determined by tissue type. These observations may suggest that multicellular organisms need a master regulator such as p53 or SOG1 that governs the majority of the transcriptional response to DNA damage to coordinate the responses of several different tissues.

## 4. What Growth Strategies Do Plants Have?

*ATM*−/− mice show pleiotropic defects (growth retardation, infertility, immune defect and high incidence of T cell lymphomas) [27], and *ATR*−/− mice show embryonic lethality [26]. As chromosome breaks are observed in both *ATM*−*/*− and *ATR*−*/*− cultured cells, the loss of genome integrity may cause the phenotype of *ATM*−*/*− and *ATR*−*/*− mice [26,113]. Moreover, mutations in some of DDR genes (ATM, BRCA1, BARD1, *etc*.) are associated with cancer formation in animals [114,115,116]. In contrast, *A. thaliana* plants with homozygous mutations in these genes show no developmental phenotype (except for partial sterility phenotype for *atm* mutants) [36,37,44,117]. Moreover, the disruption of cell-cycle control functions does not lead to tumor formation in plants [118]. How can we explain that loss of DDR components does not impair plant development or viability? Although the answer still has not been found, it may be important to consider the difference of growth strategy between animals and plants. In animals, cell lineage plays the major role in determining the organ identity, but plants can postembryonically form their organs from undifferentiated cells at meristem. Even if plants fail to develop some tissue or organs due to DDR deficiency, plants can develop new organs postembryonically [37,80]. Furthermore, there is no risk of tumor metastasis in plants because their rigid cell walls prevent cell migration. Under normal conditions, DDR mutants show no developmental phenotype, whereas, many DDR mutants show hypersensitivity toward genotoxic agents. It may be because the threshold to the activation of DDR in plants is set higher than that in animals. Additionally, plants have the option of endoreduplication in response to DNA damage. As mentioned above, endoreduplication can expand cell size by repeating DNA replication without cell division, so that even damaged cells can keep plants growing. These plant-specific features may provide less sensitivity to DDR defects. Auxins are plant hormones involved in many physiological processes, including their development [5]. It is reported that the expressions of the auxin response element *IAA5* and the auxin efflux regulator *PIN3*, which are plant-specific hormone regulators, are induced and repressed by IR, respectively, in SOG1-dependent manner [38,103]. The importance of hormone regulation is unknown, but the change in hormone levels may control plant development in response to DNA damage.

## 5. Conclusions

In this review, we have shown that many factors involved in animal DDR have been found in plant genomes based on sequence homology. It is thus clear that DNA damage sensors and the DNA repair factors are well conserved between animals and plants (Table 1). However, several key components essential for the signal transduction pathway are likely absent in plants. Although we cannot exclude the possibility that the sequence conservation is too low to identify the factors involved in the DDR pathway in plants, our findings hint that plants have unique DDR mechanisms to adapt their growth strategy. To find novel plant-specific DDR components, we have to use different methods from reverse genetics. In this regard, ongoing genetic, proteomic, and siRNA-based screens seem to provide many additional DDR components and regulators of which functions must then be defined. It is hoped that the identification of the novel mechanisms in the plant-specific DDR will be unveiled in the future.

**Table 1:** Factors involved in DNA damage response in animals and plants.

Function	Class	Animal gene	*Arabidopsis* gene	AGI code
Sensors	DSB recognition/repair	MRE11	MRE11	At5g54260
	(MRN complex)	RAD51	RAD51	At2g31970
		NBS1	NBS1	At3g02680
	ssDNA binding protein	RPA1	RPA70a	At2g06510
	PCNA-like	RAD9	RAD9	At3g05480
	(9-1-1 complex)	RAD1	RAD1	At4g17760
		HUS1	HUS1	At1g52530
	Compornet of RFC1-like	RAD17	RAD17	At5g66130
Transducers	PI3 kinase-like protein	ATM	ATM	At3g48190
		ATR	ATR	At5g40820
	PIKK binding protein	ATRIP	ATRIP	At5g45610
	Protein kinase	CHK1	Not found	
		CHK2	Not found	
Mediators	BRCT-containing	MDC1	Not found	
		53BP1	Not found	
		BRCA1	BRCA1	At4g21070
		TopBP1	MEI1?	At1g77320
	CHK1 binding	Claspin	Not found	
	Histon variant	H2AX	H2AX	At1g08880
Effectors	Transcription factor	p53	Not found	
		Not found	SOG1	At1g25580
	Cell cycle arrest	p21	Not found	
		WEE1	WEE1	At1g02970
		CDC25	CDC25-like?	At5g03455
	DNA repair	See reference [119,120]	See reference [84,85,121]	
	Apoptosis	PUMA	Not found	
		BAX	Not found	
		NOXA	Not found	

Note: AGI code: from TAIR [122].
